# Comparative Performance of Autoencoders and Traditional Machine Learning Algorithms in Clinical Data Analysis for Predicting Post-Staged GKRS Tumor Dynamics

**DOI:** 10.3390/diagnostics14182091

**Published:** 2024-09-21

**Authors:** Simona Ruxandra Volovăț, Tudor Ovidiu Popa, Dragoș Rusu, Lăcrămioara Ochiuz, Decebal Vasincu, Maricel Agop, Călin Gheorghe Buzea, Cristian Constantin Volovăț

**Affiliations:** 1 University of Medicine and Pharmacy “Grigore T. Popa” Iași, 700115 Iași, Romania; simonavolovat@gmail.com (S.R.V.); ochiuzd@yahoo.com (L.O.); deci_vas@yahoo.com (D.V.); cristian.volovat@yahoo.com (C.C.V.); 2 Faculty of Engineering, “Vasile Alecsandri” University of Bacău, 600115 Bacău, Romania; drusu@ub.ro; 3 Physics Department, Technical University “Gheorghe Asachi” Iași, 700050 Iași, Romania; m.agop@yahoo.com; 4 Clinical Emergency Hospital “Prof. Dr. Nicolae Oblu” Iași, 700309 Iași, Romania; calinb2003@yahoo.com; 5 National Institute of Research and Development for Technical Physics, IFT Iași, 700050 Iași, Romania

**Keywords:** gamma knife radiosurgery (GKRS), brain metastasis, tumor dynamics forecasting, machine learning models, autoencoders

## Abstract

**Introduction:** Accurate prediction of tumor dynamics following Gamma Knife radiosurgery (GKRS) is critical for optimizing treatment strategies for patients with brain metastases (BMs). Traditional machine learning (ML) algorithms have been widely used for this purpose; however, recent advancements in deep learning, such as autoencoders, offer the potential to enhance predictive accuracy. This study aims to evaluate the efficacy of autoencoders compared to traditional ML models in predicting tumor progression or regression after GKRS. **Objectives:** The primary objective of this study is to assess whether integrating autoencoder-derived features into traditional ML models can improve their performance in predicting tumor dynamics three months post-GKRS in patients with brain metastases. **Methods:** This retrospective analysis utilized clinical data from 77 patients treated at the “Prof. Dr. Nicolae Oblu” Emergency Clinic Hospital-Iasi. Twelve variables, including socio-demographic, clinical, treatment, and radiosurgery-related factors, were considered. Tumor progression or regression within three months post-GKRS was the primary outcome, with 71 cases of regression and 6 cases of progression. Traditional ML models, such as Logistic Regression, Support Vector Machine (SVM), K-Nearest Neighbors (KNN), Extra Trees, Random Forest, and XGBoost, were trained and evaluated. The study further explored the impact of incorporating features derived from autoencoders, particularly focusing on the effect of compression in the bottleneck layer on model performance. **Results:** Traditional ML models achieved accuracy rates ranging from 0.91 (KNN) to 1.00 (Extra Trees). Integrating autoencoder-derived features generally enhanced model performance. Logistic Regression saw an accuracy increase from 0.91 to 0.94, and SVM improved from 0.85 to 0.96. XGBoost maintained consistent performance with an accuracy of 0.94 and an AUC of 0.98, regardless of the feature set used. These results demonstrate that hybrid models combining deep learning and traditional ML techniques can improve predictive accuracy. **Conclusion:** The study highlights the potential of hybrid models incorporating autoencoder-derived features to enhance the predictive accuracy and robustness of traditional ML models in forecasting tumor dynamics post-GKRS. These advancements could significantly contribute to personalized medicine, enabling more precise and individualized treatment planning based on refined predictive insights, ultimately improving patient outcomes.

## 1. Introduction

Despite significant advances in prevention and treatment over recent decades, cancer remains a major public health issue and continues to be one of the leading causes of death worldwide [1]. The central nervous system (CNS) is frequently affected by metastases originating from systemic cancers, with the brain parenchyma being the most common site, followed by the leptomeningeal space. Parenchymal metastases and leptomeningeal disease present differently in terms of clinical symptoms, treatment approaches, and prognosis [2]. Nonetheless, it is common for these conditions to overlap: superficial brain lesions can invade the subarachnoid space, while primary leptomeningeal carcinomatosis can extend into the brain parenchyma through perivascular Virchow–Robin spaces [2,3].

Brain metastases (BM) are the most common neurological complication of cancer and the leading type of brain tumor. The precise incidence of metastatic brain tumors is not well established. Many epidemiological studies may underreport their true frequency, partly because some brain metastases are asymptomatic and partly because symptomatic lesions might be overlooked in patients with severe, advanced primary diseases [4]. Autopsy and clinical research indicate that brain metastases are found in 10–30% of adults with systemic malignancies [5,6]. Lung and breast cancers, along with melanoma and renal cell carcinoma, account for up to 75% of these metastatic brain lesions [7]. Patients often present with headaches, seizures, focal deficits, cognitive impairments, or gait disturbances, all of which significantly affect their quality of life [8]. MRI is the preferred imaging modality for detecting brain metastases due to its sensitivity, though it lacks specificity [9]. Differential diagnoses include primary brain tumors, abscesses, and vascular or inflammatory lesions. Prognosis is generally poor and is influenced by factors such as age, the extent and activity of the systemic disease, the number of brain metastases, and the patient’s overall performance status. In about half of the cases, particularly with widespread and uncontrolled systemic malignancies, death is often due to non-neural complications, and treating the cerebral disease may not significantly extend survival. For these patients, the focus is on alleviating or stabilizing neurological deficits and maintaining quality of life, typically through corticosteroids and whole-brain radiotherapy [10]. Conversely, patients with a limited number of brain metastases, good performance status, and controlled or localized systemic disease may benefit from more aggressive treatments, as addressing the brain lesions can improve both quality of life and survival. For these patients, various effective treatment options, including surgery, radiotherapy, and chemotherapy are available [11].

Recent advancements in medical technology have positioned Gamma Knife radiosurgery (GKRS) as a particularly effective method for the management of BMs, especially noted for its precision and ability to preserve surrounding healthy tissue. GKRS has become increasingly favored for its targeted approach, which is essential for the delicate neural environment. However, its application is predominantly limited by the size of the metastases; BMs exceeding 3 cm in diameter or 10 cc in volume present challenges due to heightened risks of radiation toxicity, necessitating alternative or adjunctive treatment strategies. This size constraint highlights the need for innovative approaches to enhance the efficacy of GKRS and expand its applicability [12].

Given the complexity and variability of tumor dynamics post-GKRS, there is a growing interest in leveraging advanced data analysis techniques to better predict patient’s outcome. Autoencoders (AE), a form of neural networks, along with other machine learning (ML) models, have shown promise in deciphering the intricate patterns in clinical data associated with tumor response to treatment. These models offer potential breakthroughs in precision medicine by enabling more personalized treatment plans based on predicted tumor behavior [13,14].

Machine learning (ML), a branch of artificial intelligence (AI), employs algorithms designed to automatically identify patterns within data and use these patterns to make predictions [15]. These algorithms have recently been developed as support tools for enhancing medical decision-making, particularly in the diagnosis, prognosis, and treatment of various diseases [16]. However, their widespread adoption faces several challenges [17,18,19,20,21]. Notably, ML techniques have been applied in the study of neurological and brain diseases. For instance, Uspenskaya-Cadoz et al. [22] utilized logistic regression (LR), decision trees (ET), random forests (RF), and gradient-boosted trees (GBT) for diagnosing Alzheimer’s disease (AD). Similarly, Ghafouri-Fard et al. [23] employed artificial neural networks (ANNs) to predict the risk of multiple sclerosis (MS) based on genetic profiles.

To evaluate the pairwise differences in predictive accuracy among the 6 machine learning models, we employed McNemar’s test. This non-parametric method is used to compare the performance of two related classification models on the same dataset. Given the multiple comparisons across model pairs, we applied Bonferroni and False Discovery Rate (FDR) corrections to adjust for the increased risk of Type I errors [24,25,26].

An autoencoder (AE) is a special type of neural network that is trained to copy its input to its output. It consists of two main components: an encoder and a decoder. The encoder compresses the input data into a smaller, dense representation, while the decoder tries to reconstruct the original input from this compressed version [27,28,29,30]. After training, only the encoder is retained, and the decoder is discarded. The encoder can then be employed for feature extraction on raw data, which can be used to train other machine learning models [31]. Even if autoencoders are not a particularly new method in deep learning (DL), they have evolved significantly over time. They were first introduced in the 1980s, but their use became more prominent with the rise of deep learning techniques in the 2000s and 2010s [32].

Autoencoders present distinct benefits compared to traditional feature selection techniques, particularly in their capacity to capture non-linear relationships between input features and create compressed data representations. Nonetheless, they also have certain drawbacks, such as a tendency to overfit and challenges in assessing feature importance. Despite these limitations, autoencoders remain a powerful tool for machine learning professionals. By adopting a well-designed architecture and incorporating regularization methods, it is possible to mitigate these issues and achieve effective outcomes in feature selection and dimensionality reduction.

Predicting tumor dynamics after Gamma Knife radiosurgery (GKRS) is essential for personalizing treatment in brain metastases (BMs). Traditional machine learning (ML) models, while useful, often depend on manual feature selection and may not handle complex or high-dimensional data effectively. Autoencoders, on the other hand, automatically learn and compress data features, capturing intricate patterns that traditional methods might miss. This capability makes them particularly suited for enhancing predictive accuracy. By integrating autoencoder-derived features with conventional ML algorithms, this study explores whether such hybrid approaches can improve predictions and contribute to more personalized treatment plans for patients with BMs.

Major contributions of the paper may be summarized as follows:

- Autoencoder Evaluation: Investigates the use of autoencoders for feature extraction in predicting tumor dynamics.
- Model Comparison: Compares traditional ML models with those enhanced by autoencoder features.
- Accuracy Improvement: Shows how integrating autoencoders improves predictive accuracy.
- Personalized Medicine Impact: Highlights potential benefits for personalized treatment strategies.

The paper is structured as follows:

- Section 2: Materials and Methods, covers GKRS implementation, dataset preparation, model descriptions, and training/validation.
- Section 3: Results and Discussion, details performance metrics, comparative analysis, and interpretation.
- Section 4: Conclusion, summarizes findings and implications for personalized medicine.

This paper aims to explore and compare the performance of new DL autoencoders and traditional machine learning models in analyzing clinical datasets for predicting tumor dynamics following staged GKRS. By focusing on such predictive models, this study seeks to contribute to the broader efforts of improving treatment outcomes and patient quality of life through more informed clinical decisions.

## 2. Materials and Methods

In this study, we present a comprehensive outline of our research framework, including the methodologies employed and the characteristics of the participants involved.

**Figure 1:**
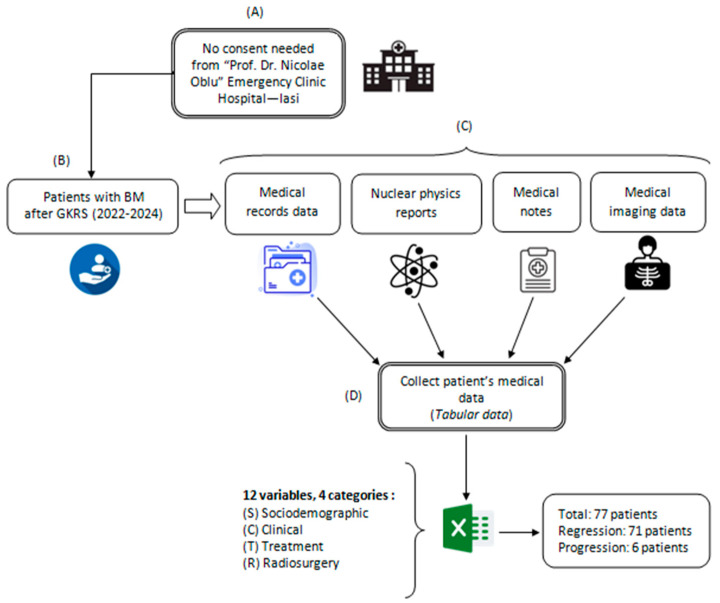
Data collection process: (**A**) consent; (**B**) patient selection; (**C**) data extraction;(**D**) data tabulation.

The investigation contains a retrospective analysis of medical records for patients who underwent Gamma Knife Radiosurgery (GKRS) using mask-based fixation for brain metastases originating from various primary cancers. This analysis covered treatments conducted between July 2022 and March 2024 at the “Prof. Dr. Nicolae Oblu” Emergency Clinical Hospital in Iasi. Data were gathered from multiple medical sources, following the procedure depicted in Figure 1. All experimental protocols adhered to applicable guidelines and regulations. As the study exclusively utilized existing medical data, patient consent was not required, and due to its retrospective nature, approval from the Ethics Committee of the “Prof. Dr. Nicolae Oblu” Emergency Clinical Hospital in Iasi was not necessary.

The variables that were collected from patient data were considered as input data (predictors) and were grouped into 4 categories: socio-demographic (S), clinical (C), treatment (T) and radiosurgery (R). The variables for patients’tumor dynamics (progression or regression within 3 months following GKRS treatment) were considered as output data (response).

### 2.1. Approach to Gamma Knife Radiosurgery Implementation

This section outlines the systematic approach employed for administering Gamma Knife radiosurgery (GKRS), with a focus on the critical planning and execution phases that are essential for effective treatment. All patients received GKRS using the Leksell Gamma Knife ICON (Elekta AB, Stockholm, Sweden). MRI scans were conducted using a 1.5 Tesla whole-body scanner (GE SIGMA EXPLORER) equipped with a 16-channel head coil. The MRI protocol consisted of two main components: Firstly, the conventional anatomical MRI (cMRI) protocol, which is standard for clinical diagnosis of brain tumors, included sequences such as axial fluid-attenuated inversion recovery (FLAIR) and high-resolution contrast-enhanced T1-weighted (CE T1w). Secondly, the advanced MRI (advMRI) protocol, an extension for clinical diagnosis, incorporated axial diffusion-weighted imaging (DWI) with b values of 0 and 1000 s/mm^2^, and gradient echo dynamic susceptibility contrast (GE-DSC) perfusion MRI, performed with 60 dynamic measurements during the administration of gadoterate meglumine at 0.1 mmol/kg body weight.

All MRI images were registered using Leksell Gamma Plan (LGP, Version 11.3.2, TMR algorithm), with any images showing motion artifacts being excluded from the analysis. Tumor volumes were calculated by the LGP without applying a margin. Typically, a total dose of 30 Gy, delivered in three stages of GKRS, was prescribed based on the linear quadratic model [33,34] and supported by previous studies, including Higuchi et al. (2009) [35]. The GKRS treatment plan was developed through a collaborative decision-making process involving a neurosurgeon, radiation oncologist, and medical physicist.

### 2.2. Dataset

The study included 77 patients (45 males and 32 females) with ages ranging from 39 to 85 years, with a median age of 64. All participants had been previously diagnosed with brain metastases (BMs). The general characteristics of the patient cohort, including age, sex, tumor volume at one-year control (C1yr), presence of extracranial metastases (MTS extracranial), pretreatment history, survival status within one year, Karnofsky performance scale (KPS) score, number of lesions, beam-on time relative to the number of isocenters for each of the three volumes treated, total tumor volume, and tumor dynamics (progression or regression within three months post-GKRS), are detailed in Table 1.

The dataset utilized in this study is structured as a table consisting of 77 records (rows) and 13 variables (columns). Each row corresponds to an individual patient’s data, while the columns represent the specific variables analyzed in the study. Of these, 12 variables were treated as input variables (independent variables) for the system. The final column serves as the output variable (dependent variable), which indicates tumor dynamics. Specifically, this output variable captures the number of patients who experienced tumor regression (denoted as cured = 0) or tumor progression (denoted as not cured = 1).

#### 2.2.1. Data Preprocessing

During the process of labeling medical data, we extracted pertinent features from the extensive information available within the electronic medical record (EMR) system. A common challenge in applying machine learning (ML) to EMR data is handling incomplete datasets. To address this issue, two strategies can be employed: imputation and exclusion of missing data. Imputation Methods: To handle missing data, categorical variables were imputed with the most recent valid value from the preceding row. Numerical variables with missing values were filled using the median value of the dataset, which robustly represents the central tendency of the data. Transformation Techniques: Categorical variables were encoded using label encoding to convert them into numerical format. Numerical features were normalized using min-max scaling, which rescales values to a range between 0 and 1, ensuring uniformity in the data distribution [36,37].

**Figure 2:**
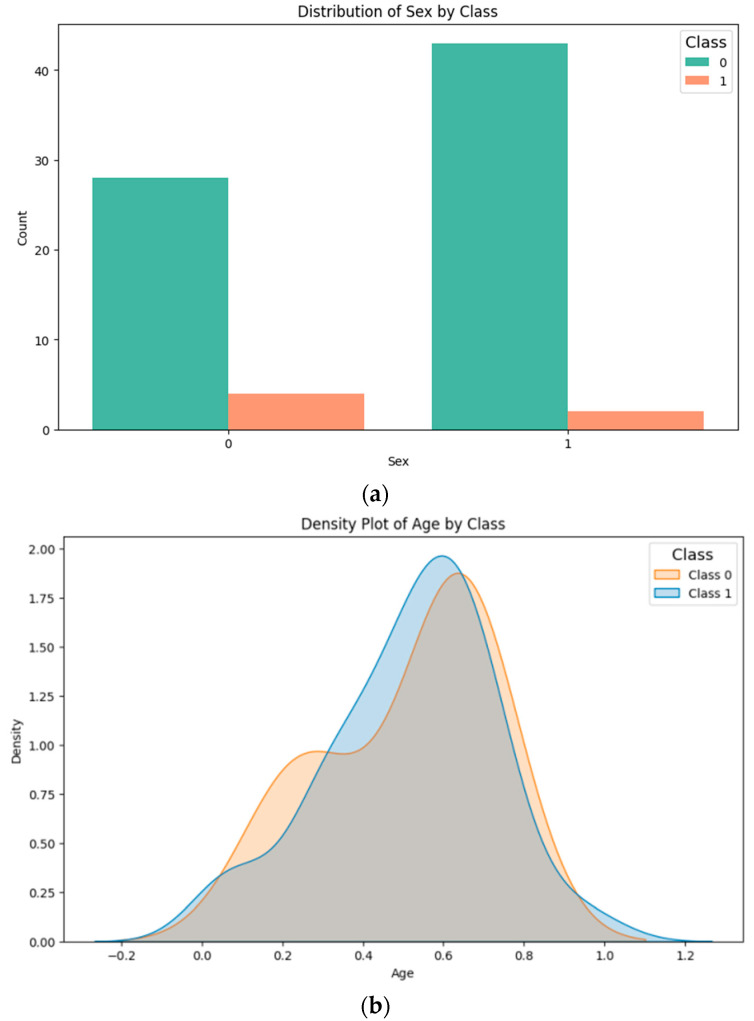
Analysis of variables in the dataset: (**a**) sex variable; (**b**) age variable.

Given the medical context of this study, it was crucial to examine the data for potential confounding variables that could negatively impact the predictive outcomes [38]. To this end, we investigated the possible confounding effects of sex and age. For the categorical variable of sex, we conducted a chi-square test of homogeneity to determine if the distribution of men and women across the data groups was statistically significant, finding no significant difference (*p*-value = 0.385; Figure 2a). Regarding the age variable, a Student’s *t*-test was applied to assess whether there was a statistically significant age difference between the groups (class 0: mean = 51.6; class 1: mean = 51.1), and again, no significant difference was observed (*p*-value = 0.955; Figure 2b).

Based on our analysis, we determined that the variables age and sex did not function as confounding factors, allowing us to proceed with data preprocessing. Additionally, we conducted a correlation analysis of the 12 independent variables. Cramér’s V test [39] was utilized to assess the linear correlation between categorical variables, while Pearson’s correlation coefficient was applied to the numerical variables. A threshold value of 0.7 or higher was used to identify significant positive or negative correlations [40]. The calculations were performed using the ‘dython’ library [41] available in the Python programming language. As a result of this analysis, none of the 12 independent variables were excluded, leading to a final dataset comprising 12 independent variables and 1 dependent variable, which were subsequently used in the machine learning system proposed in this study.

Consequently, the final dataset, consisting of 77 records with 12 independent variables and 1 dependent variable, was split into two subsets: 70% (*n* = 54) for training and validating the machine learning (ML) model, and 30% (*n* = 23) for testing.

#### 2.2.2. Data Balancing

**Figure 3:**
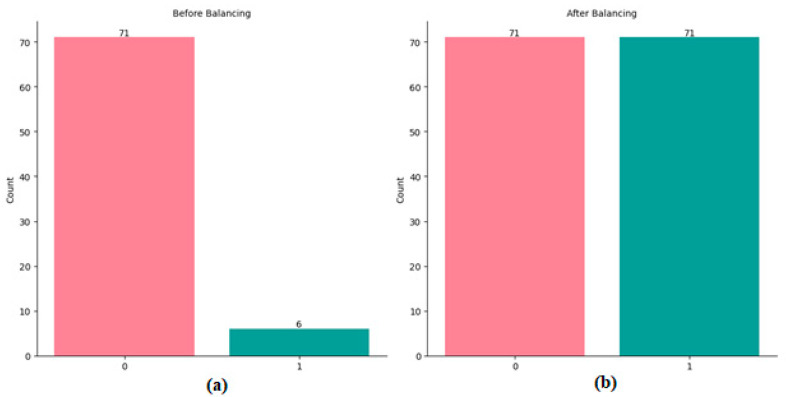
Number of records for each class: (**a**) before and (**b**) after data balancing.

The original training dataset exhibited an imbalance with respect to the dependent variable, tumor dynamics, with 71 records classified as class 1 and only 6 records as class 0. To address this imbalance, we applied the Synthetic Minority Oversampling Technique (SMOTE), which generates new synthetic instances of the minority class rather than simply duplicating existing ones [42,43]. After applying SMOTE, the dataset was balanced, resulting in a total of 142 records, with 71 records for each class, as illustrated in Figure 3.

Finally a balanced training dataset made up of 142 records, was obtained.

### 2.3. Models Description

In this study, our preference for the Python 3.10.12 programming language from the Google Colab environment was because Google Colab offers a powerful, accessible, and collaborative environment for Python programming, particularly suited for data-intensive and computational tasks [44]. The project utilized various open-source Python 3.x libraries to enhance its functionality. NumPy was employed for numerical data manipulation, while pandas facilitated data structuring and analysis. For data visualization, Matplotlib and seaborn were used. Machine learning, deep learning, and data mining tasks were carried out using TensorFlow, Keras, and scikit-learn. Also, we used LazyPredict which provides a quick and straightforward way to benchmark multiple classification models with default settings, helping to identify the most promising algorithms for further tuning and optimization.

#### 2.3.1. Traditional ML Models

**Figure 4:**
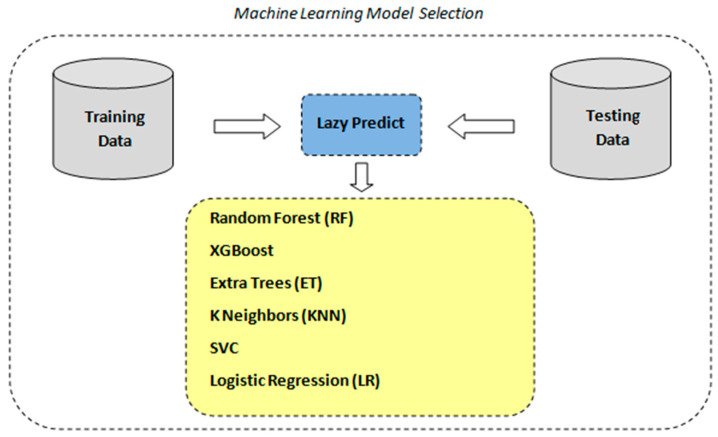
ML model selection with lazy predict.

As stated above, in order to identify the optimal machine learning model for our classification task, we utilized the LazyPredict tool to automatically fit and evaluate multiple standard classifiers with their default parameters according to the scheme depicted in Figure 4. LazyPredict facilitated an expedient comparative analysis across various models based on standard performance metrics (see Table 2).

The analysis demonstrates a wide variance in performance and efficiency among the models. SVC, Extra Trees, and KNN show the highest accuracy, indicating strong separability and robustness in the dataset. Models like Logistic Regression and XGBoost, while still effective, indicate some limitations under default settings. The Random Forest Classifier, despite being an ensemble method known for its high accuracy in many scenarios, appears less effective in this specific case, suggesting a potential need for parameter tuning or a different modeling approach.

#### 2.3.2. Autoencoders for Feature Extraction

**Figure 5:**
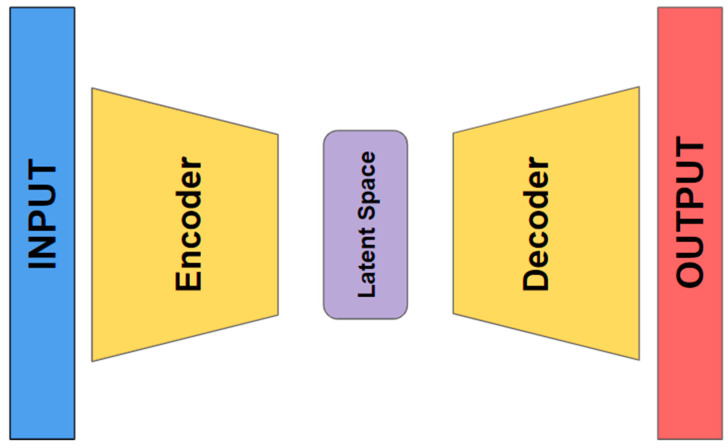
Autoencoder general architecture.

Autoencoders are a specialized type of neural network that are designed to learn efficient representations of input data. They achieve this by compressing the input data into a lower-dimensional latent space and then reconstructing the original data from this compressed representation (refer to Figure 5).

In our study, an autoencoder was trained to capture the underlying patterns and important features of the clinical dataset. Once trained, the encoder part was separated, saved, and used as features input for thetraditional machine learning algorithms selected. This preprocessing step aimed to enhance the models’ ability to predict tumor dynamics post-treatment, providing a comparative analysis of the performance improvements achieved through autoencoder-based feature extraction.

We used 2 types of autoencoders’ architecture:

- With no compression in the bottleneck layer
  **Encoder:**
    - An input layer is defined.
    - The first encoder layer has twice the number of input features, followed by batch normalization and LeakyReLU activation.
    - The second encoder layer has the same number of input features, followed by batch normalization and LeakyReLU activation.
  **LatentSpace(Bottleneck):**
    - The latentspace has the same number of neurons as the input features (no compression).
  **Decoder:**
    - The first decoder layer has the same number of input features, followed by batch normalization and LeakyReLU activation.
    - The second decoder layer has twice the number of input features, followed by batch normalization and LeakyReLU activation.
  **Output layer:**
    - The output layer has the same number of input features with a linear activation function.
- With compression in the bottleneck layer
  **Encoder:**
    - An input layer is defined.
    - The first encoder layer has twice the number of input features, followed by batch normalization and LeakyReLU activation.
    - The second encoder layer has the same number of input features, followed by batch normalization and LeakyReLU activation.
  **LatentSpace (Bottleneck):**
    - The latentspace has half the number of neurons as the input features (compressed size).
  **Decoder:**
    - The first decoder layer has the same number of input features, followed by batch normalization and LeakyReLU activation.
    - The second decoder layer has twice the number of input features, followed by batch normalization and LeakyReLU activation.
  **Output layer:**
    - The output layer has the same number of input features with a linear activation function.

### 2.4. Training and Validation

#### 2.4.1. Traditional ML Models

**Figure 6:**
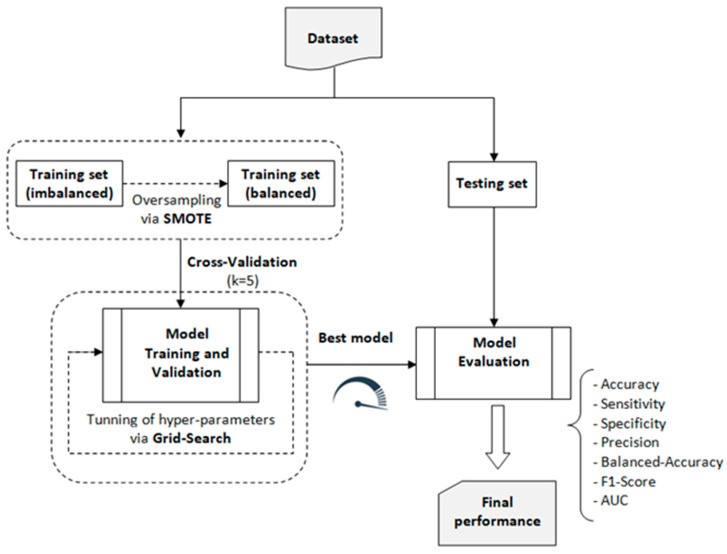
Process used to train the chosen traditional ML models.

For the construction, validation, and evaluation of the traditional ML system, we followed the process outlined in Figure 6. This process involved using the balanced training dataset to build and validate six machine learning models: Random Forest (RF), Extreme Gradient Boosting (XGB), Extra Trees (ET), k-Nearest Neighbors (KNN), Support Vector Classifier (SVC), and Logistic Regression (LR). These models were selected using the Lazy Predict library. Based on the performance metrics obtained from this validation process, the model with the best overall performance was selected for further analysis and application.

In the training phase, the grid search technique [45] was used to find the optimalhyperparameters of the traditional ML models. The set of search valuesdefined for the hyperparameters is given in Table 3.

**Figure 7:**
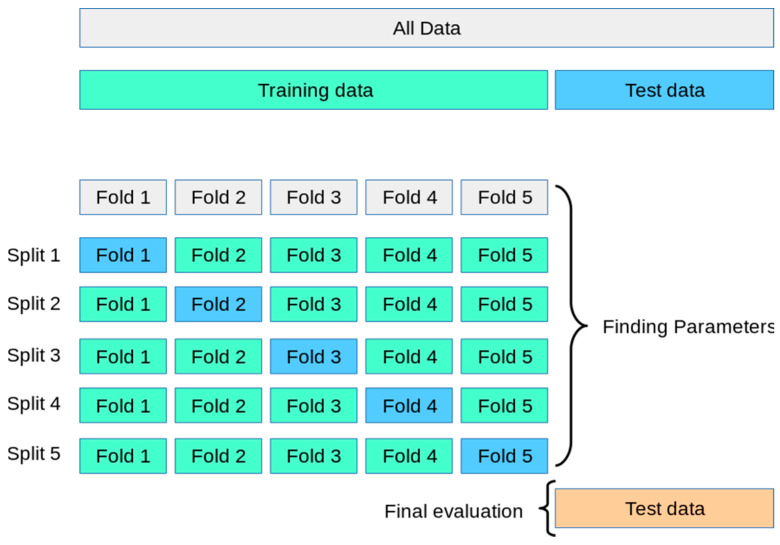
5-fold cross-validation.

During the training process, we employed a resampling technique as illustrated in Figure 7. The training dataset was divided into five subsets, where one subset was used for validation and the remaining four subsets were used for training. This approach followed the 5-fold cross-validation method, a widely used technique for selecting and validating machine learning models [46,47].

#### 2.4.2. Autoencoders

**Figure 8:**
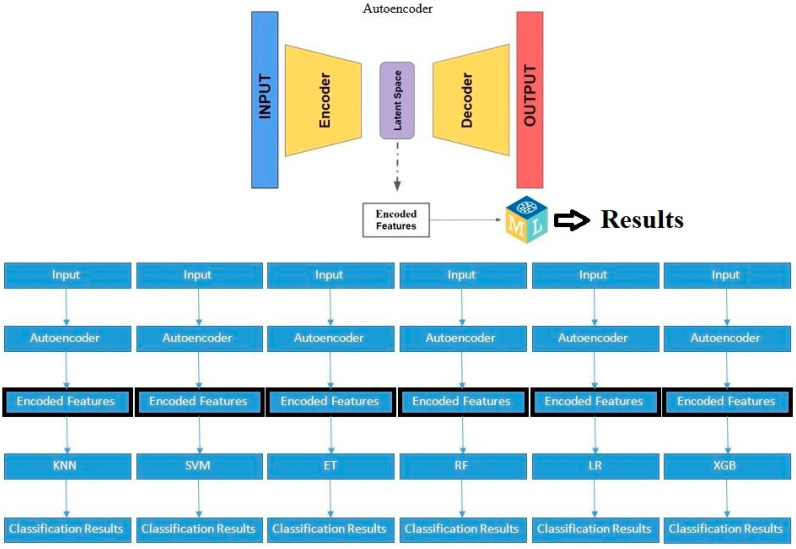
The hybrid autoencoder—based machine learning classifier.

The autoencoder model *with no compression in the bottleneck layer* is compiled with Adam optimizer, mean squared error (MSE) loss, and accuracy as the metric. The autoencoder is trained to reconstruct the input using the training data, and the loss is validated on the testing data. A separate encoder model (without the decoder) is defined to extract the compressed features. The trained encoder model is saved to a file. The training and testing data are encoded using the trained encoder model. A pipeline of ML classifiers(KNN, SVM, ET, RF, LR, XGB) is trained on the encoded training data. The classifier’s performance is evaluated on the encoded testing data using classification metrics (accuracy, precision, recall, F1-score) (see Figure 8). The same process is repeated for the autoencoder model *with compression in the bottleneck layer*.

KNN: K-nearest neighbor; SVM: Support Vector Machine; ET: Extra Trees; RF: Random Forrest; 

LR: Logistic Regression; XGB: XGBoost.

**Figure 9:**
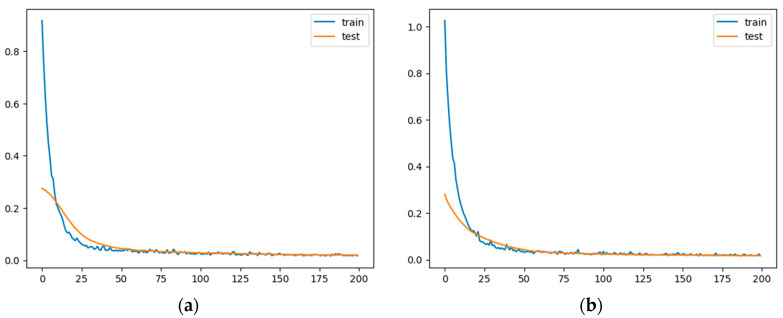
Training and validation loss for the autoencoders (horizontal axis the number of Epochs, vertical axis the value of the training and validation loss). (**a**) with no compression in the bottleneck layer; (**b**) with compression in the bottleneck layer.

The training and validation loss are plotted to visualize the models’ performance over epochs (see Figure 9).

## 3. Results and Discussion

### 3.1. Performance Metricsfor Traditional ML

**Figure 10:**
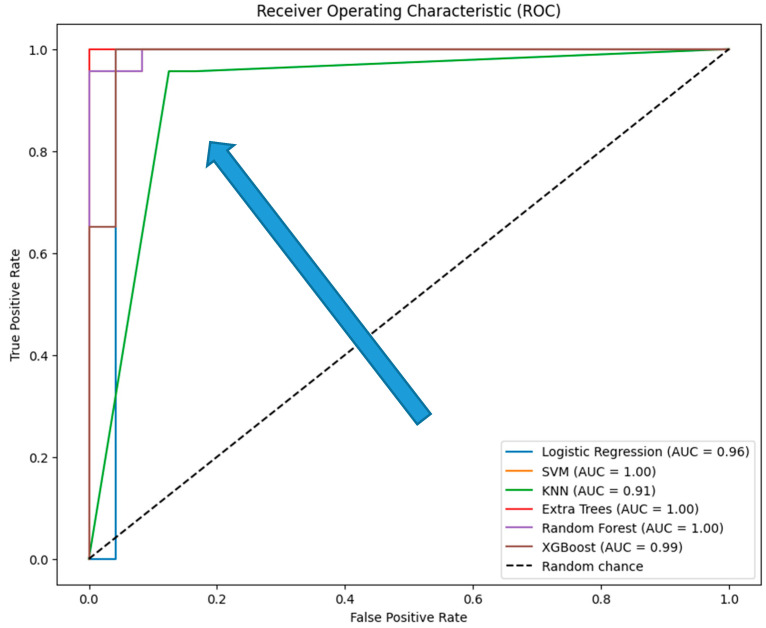
ROC curves for the six traditional ML models tested, with hyperparameter tuning.

**Figure 11:**
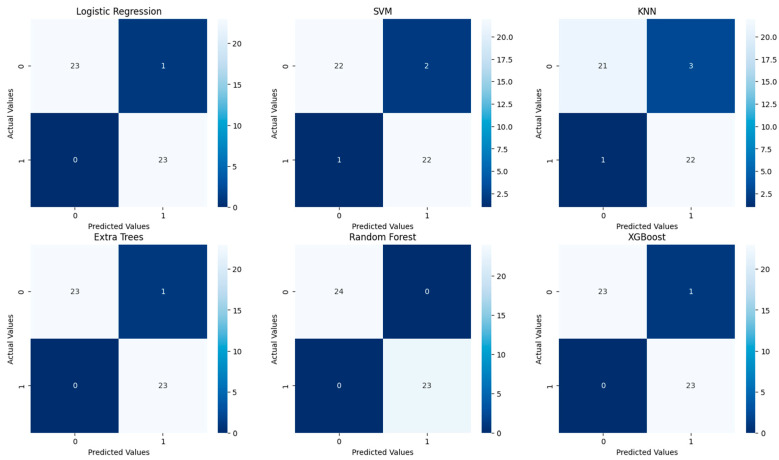
Confusion matrices for the six traditional ML models tested, with hyperparameter tuning.

The evaluation of the six traditional machine learning (ML) models with hyperparameter tuning reveals insightful performance metrics across various classification techniques. The models tested include Logistic Regression, Support Vector Machine (SVM), K-Nearest Neighbors (KNN), Extra Trees, Random Forest, and XGBoost. These models were evaluated based on the classification report (precision, recall, F1-score, and accuracy) (see Table 4), ROC curves (Figure 10), and confusion matrices (Figure 11), with specific attention to accuracy and AUC (Area Under the Curve) metrics (Table 5).

Both Logistic Regression and XGBoost exhibit strong performance, achieving an accuracy of 0.98 and F1-scores close to 0.98 for both classes, reflecting a good balance between precision and recall. In contrast, SVM and Random Forest models demonstrate slightly lower performance, with accuracies of 0.94 and 0.96, respectively. While their F1-scores are balanced, SVM shows a minor drop in recall for class 0, indicating potential underfitting for that class. KNN, with an accuracy of 0.91, performs less effectively, particularly struggling with class 0, suggesting challenges in handling class imbalance or data complexity. The Extra Trees model, with perfect scores across all metrics, indicates potential overfitting, raising concerns about its ability to generalize to new data.

The ROC curves which provide a visual representation of the true positive rate (sensitivity) versus the false positive rate (1-specificity) across different threshold settings, demonstrate that XGBoost and Logistic Regression achieve near-perfect performance, with AUC values close to 1.0, indicating excellent class distinction. Extra Trees and Random Forest also perform strongly, with Extra Trees showing a steep rise in the curve at low false positive rates due to its perfect classification. SVM, while still effective, has a slightly lower AUC, reflecting its relatively reduced recall. KNN shows a less steep ROC curve, indicating lower sensitivity and a correspondingly lower AUC, consistent with its overall weaker performance.

Extra Trees and XGBoost are top performers, with perfect or near-perfect accuracy and AUC values; however, Extra Trees may be overfitting, while XGBoost’s strong performance is more reliable due to its regularization techniques. Logistic Regression also performs excellently, offering a strong balance of interpretability and accuracy. SVM and Random Forest are reliable, with good accuracy and AUC, but slightly lag behind the top models. KNN, though it shows the lowest performance, still maintains a reasonable balance between accuracy and AUC, but is less competitive overall.

Confusion matrices reveal the alignment between predictions and actual outcomes. Logistic Regression and XGBoost likely show very few misclassifications, with most instances correctly identified. Extra Trees would display perfect classification, with no errors, raising concerns about overfitting. SVM and Random Forest might show a few misclassifications, particularly in SVM, which could struggle more with the minority class. KNN’s confusion matrix would reflect its lower accuracy, likely with more frequent misclassifications between class 0 and class 1.

The McNemar’s test results and Bonferroni/FDR corrections provide insights into model performance differences:

**McNemar’s Test Results:**

McNemar’s test assesses if two models differ in classification results. A *p*-value < 0.05 suggests significant differences.

Most model pairs have *p*-values > 0.05, indicating no significant differences. Examples:

▪ Logistic Regression vs. SVM (*p* = 0.617)
▪ Logistic Regression vs. KNN (*p* = 0.371)
▪ Logistic Regression vs. Extra Trees (*p* = 1.0)
▪ Logistic Regression vs. XGBoost (*p* = 0.0)—This is the only pair showing a significant difference.

**Bonferroni and FDR Corrections:**

- After Bonferroni and FDR corrections, most *p*-values remain at 1.0, indicating no significant differences, except for **Logistic Regression vs. XGBoost** (*p* = 0.0), which remains significant.

**Finally:**

- Logistic Regression and XGBoost are the only models with significantly different performance (*p* = 0.0). For all other pairs, the performance is statistically similar.

In conclusion, XGBoost and Logistic Regression emerge as the top-performing models, excelling in both accuracy and generalization, as indicated by their ROC and AUC scores. Extra Trees also performs exceptionally but raises concerns about overfitting. SVM and Random Forest are reliable alternatives, though slightly less competitive, while KNN trails behind. The final model selection should account for not only these performance metrics but also the specific application’s requirements for interpretability, computational efficiency, and robustness to overfitting.

### 3.2. Performance Metrics in Autoencoders for Feature Extraction

**Figure 12:**
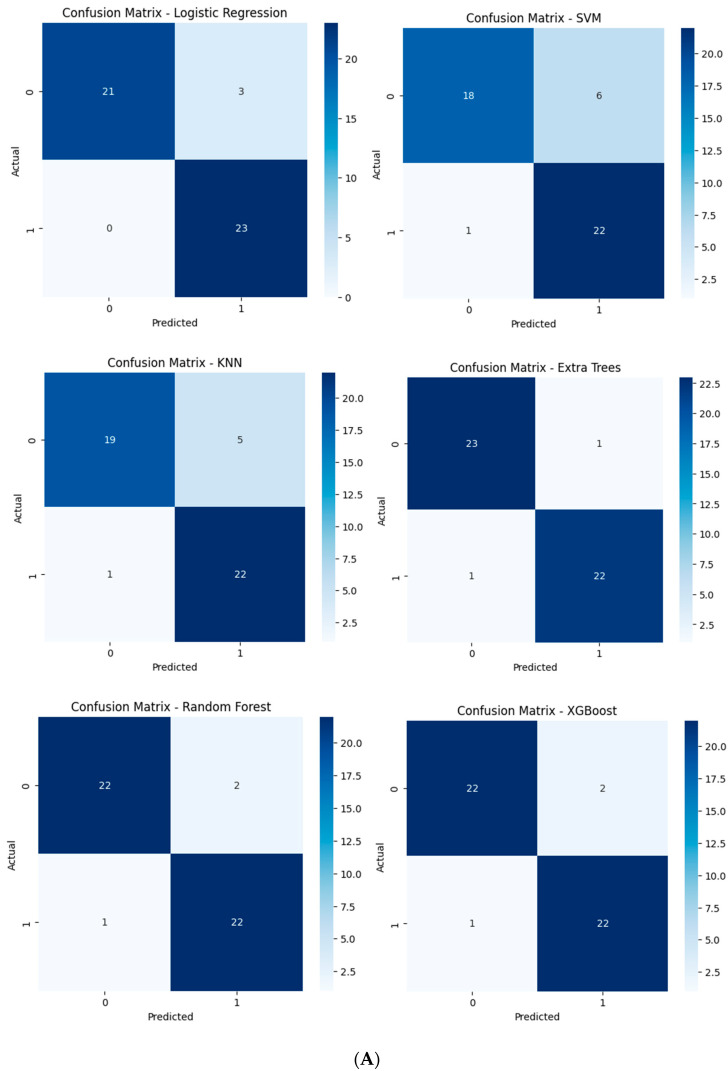

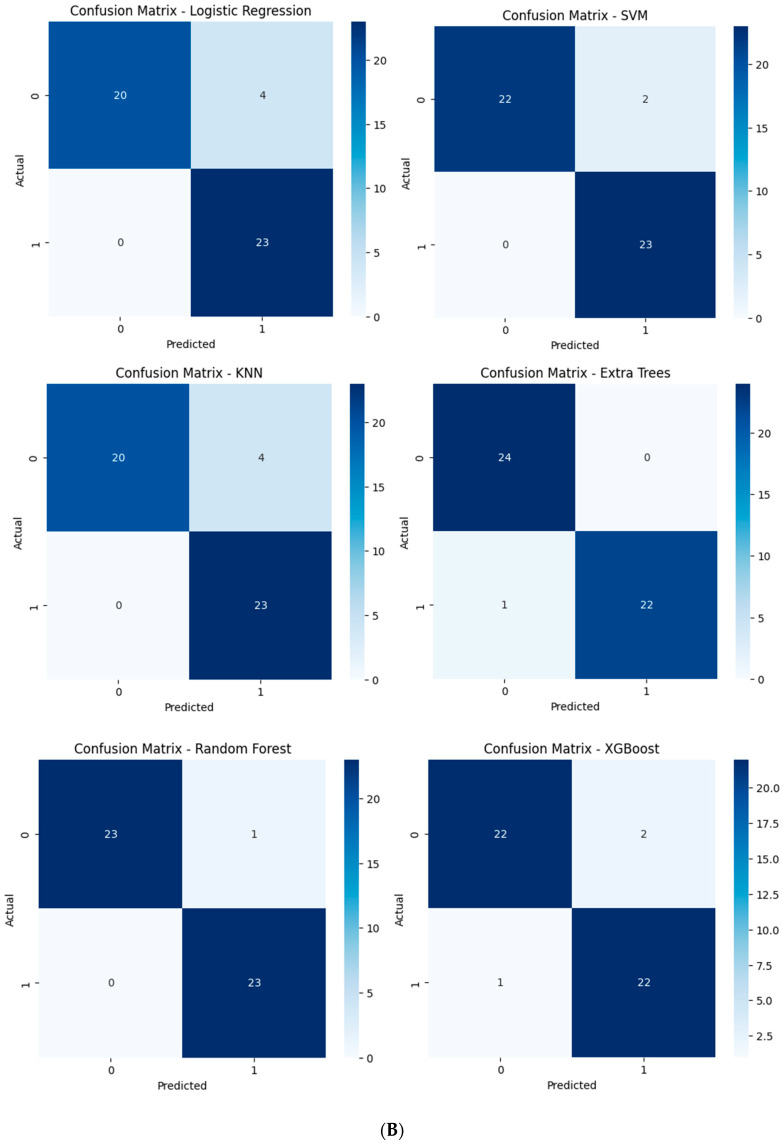
Confusion matrices for the six models tested using the encoder from the AE without compression (**A**) and with compression (**B**) in the bottleneck layer.

**Figure 13:**
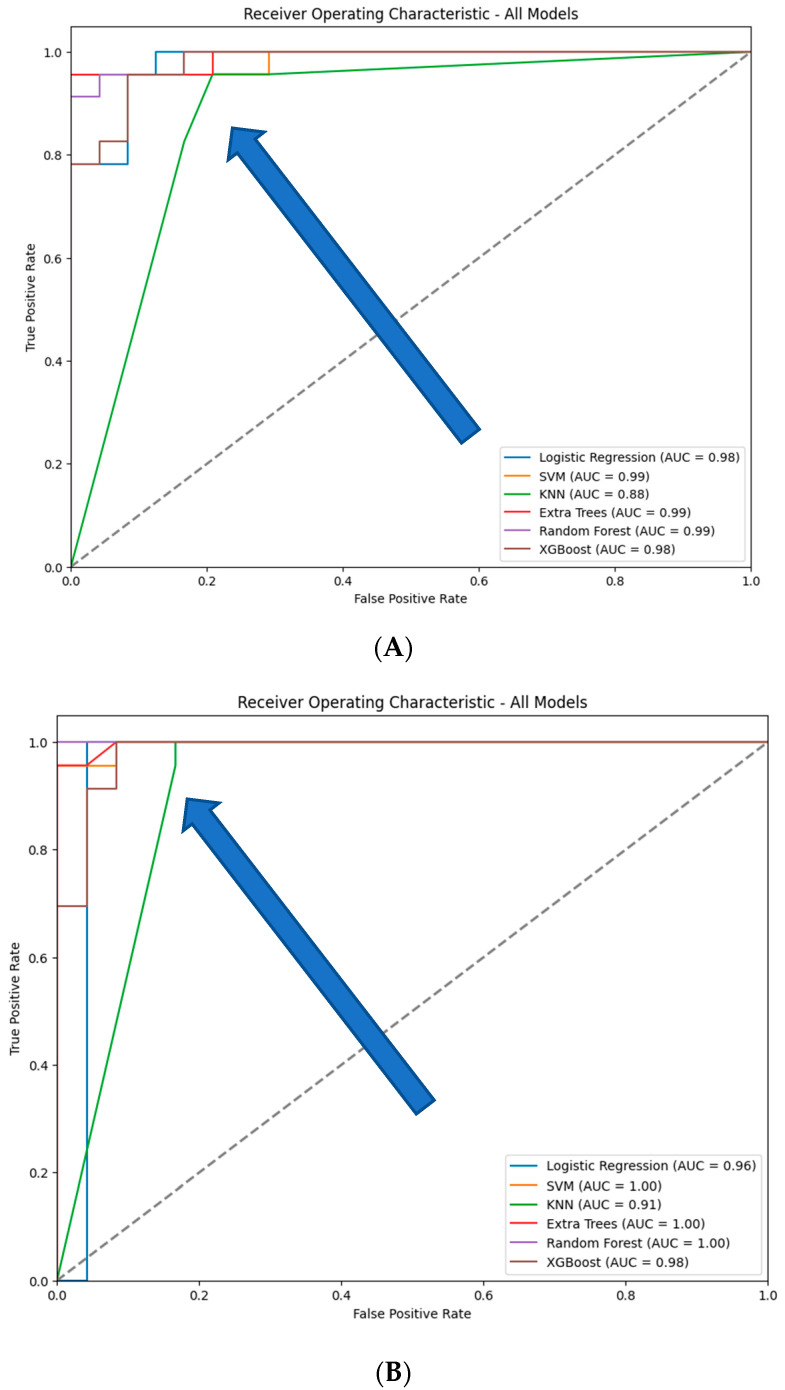
ROC curves for the six models tested using the encoder from the AE without compression (**A**) and with compression (**B**) in the bottleneck layer.

This analysis compares the performance of six traditional machine learning models-Logistic Regression, SVM, KNN, Extra Trees, Random Forest, and XGBoost—when using features extracted by an autoencoder’s encoder. The performance metrics include classification reports (precision, recall, F1-score, and accuracy) (Table 6), confusion matrices (Figure 12), ROC curves (Figure 13), accuracy, and AUC, focusing on scenarios with and without compression in the bottleneck layer.

With feature compression, most models show improved performance:

- Logistic Regression: Accuracy of 0.94 with balanced precision, recall, and F1-scores around 0.94. Without compression, accuracy drops to 0.91, with recall for class 0 at 0.83 and precision for class 1 at 0.85.
- SVM: Accuracy of 0.96 with compression, with balanced precision and recall around 0.96. Without compression, accuracy drops to 0.85, with recall for class 0 falling to 0.75.
- KNN: With compression, accuracy is 0.91, with F1-scores close to 0.92. Without compression, accuracy decreases to 0.87, with lower precision and recall for class 0.
- Extra Trees: Slightly better performance with compression, achieving 0.98 accuracy. Without compression, accuracy is slightly lower at 0.96.
- Random Forest: Best performance with compression at 0.98 accuracy. Without compression, accuracy is slightly lower at 0.94.
- XGBoost: Stable performance with or without compression, maintaining an accuracy of 0.94.

Overall, accuracy and AUC are generally higher with compression, especially for SVM and Random Forest, indicating better performance with more informative, reduced features. Without compression, while still robust, models show slightly lower accuracy and AUC, suggesting that the additional features might introduce noise or redundancy, slightly hindering their performance.

With feature compression, confusion matrices indicate fewer misclassifications for models like SVM, Extra Trees, and Random Forest, confirming their higher precision and recall. The compressed feature space leads to clearer predictions. Without compression, more misclassifications are evident, particularly for SVM and KNN, reflecting their struggles with increased dimensionality and potential noise in the uncompressed features.

With compression, most models, particularly SVM, Extra Trees, and Random Forest, exhibit stronger ROC curves and higher AUC values, indicating improved class discrimination. Compression likely reduces noise or irrelevant features, resulting in clearer decision boundaries. Without compression, models like SVM and KNN show a noticeable decline in their ROC curves, reflecting reduced sensitivity and specificity, likely due to the added complexity of uncompressed features.

The analysis demonstrates that compression in the bottleneck layer of the autoencoder generally enhances the performance of traditional machine learning models. SVM, Logistic Regression, and KNN benefit most, showing significant improvements in accuracy, precision, recall, and AUC. Extra Trees and Random Forest also perform better with compression, though they are more robust to feature representation changes. XGBoost maintains consistent performance across both compressed and uncompressed features, highlighting its adaptability. Overall, using compressed autoencoder-generated features improves model performance by enhancing feature quality and reducing noise.

### 3.3. Comparative Analysis

Based on the data provided from both traditional machine learning (ML) models and deep learning (DL) autoencoders for predicting tumor dynamics following staged GKRS, here is a comparative analysis of their performance:

**Traditional Machine Learning Models:** The traditional ML models, specifically Logistic Regression, SVM, KNN, Extra Trees, Random Forest, and XGBoost, showed a range of accuracies and metrics. Extra Trees stood out with perfect scores across precision, recall, F1-score, and accuracy, suggesting potential overfitting. Logistic Regression and XGBoost also showed high performance with accuracies and F1-scores around 0.98. Meanwhile, KNN lagged with the lowest accuracy and F1-scores around 0.91, indicating less robustness in handling the data complexity or class imbalances.

**Deep Learning Autoencoders:** The use of features extracted by an autoencoder’s encoder in the same set of traditional ML models showed varying performances depending on whether compression was applied in the bottleneck layer. Generally, models with compression in the bottleneck layer showed higher accuracy, precision, recall, and AUC, indicating better handling of the feature space by focusing on the most informative aspects of the data. SVM, Logistic Regression, and KNN particularly benefited from compression, showing marked improvements in all metrics.

**Comparative Insights:**

- Accuracy and Generalization: Both Logistic Regression and XGBoost excelled in both setups, but with the autoencoder, there was generally an enhancement in model performance when compression was applied. This suggests that the dimensionality reduction and noise filtering provided by the bottleneck layer can enhance the classification effectiveness.
- Handling of Features: Autoencoders with compression seem to help models like SVM and KNN, which otherwise show decreased performance in class separation and sensitivity when uncompressed features are used. This highlights the strength of DL techniques in extracting and utilizing compact, informative features from complex datasets.
- Potential Overfitting: Extra Trees showed perfect classification in the traditional setup, which might not generalize well to new data, a typical indication of overfitting. However, with the use of autoencoders, even though performance remained high, there was a slight reduction in some metrics, suggesting a more realistic generalization capability.
- Robustness: XGBoost maintained consistent performance across both uncompressed and compressed scenarios, reflecting its robustness due to inherent regularization capabilities. This adaptability makes it a strong candidate for clinical datasets where robustness to feature variations is critical.

In conclusion, the integration of DL techniques, specifically autoencoders, with traditional ML models can enhance the predictive performance on clinical datasets by improving the quality of features used for model training. This hybrid approach appears particularly beneficial in applications where data complexity and dimensionality pose significant challenges. Models like SVM, Logistic Regression, and XGBoost benefit noticeably from such integration, suggesting a promising direction for future research and application in clinical data analysis and tumor dynamics prediction.

### 3.4. Discussion

**Overview of Findings**: This study examined the performance of traditional machine learning (ML) models—Logistic Regression, SVM, KNN, Extra Trees, Random Forest, and XGBoost—both independently and in combination with features extracted from deep learning autoencoders. The results suggest that incorporating autoencoder-extracted features, particularly with compression applied at the bottleneck layer, can significantly enhance the accuracy and robustness of these models. This aligns with recent trends in medical data analysis, where hybrid approaches that integrate deep learning with traditional ML techniques have shown promise in tackling complex predictive tasks, such as forecasting tumor dynamics following Gamma Knife Radiosurgery (GKRS) [48].

**Comparison with Previous Studies**: The enhanced performance observed in models like SVM and KNN when using compressed features is consistent with findings from previous research. Autoencoders, by reducing dimensionality and filtering noise, can extract the most informative aspects of the data, leading to improved model performance. For instance, studies have demonstrated the effectiveness of autoencoders in improving the classification accuracy of ML models in various medical applications, including image recognition and genetic data analysis [49,50]. Specifically, the utility of autoencoders in handling complex datasets has been documented in neurological research, where they have been employed to enhance the prediction of diseases such as Alzheimer’s and Parkinson’s [51]. XGBoost’s strong performance in both standalone and hybrid configurations is also supported by the literature. Known for its robustness and ability to handle large and complex datasets, XGBoost has been widely adopted in medical research for tasks such as risk prediction and survival analysis. Its regularization capabilities help prevent overfitting, making it an ideal choice for clinical datasets, which often contain noise and high variability [52,53]. This model’s consistent performance underscores its potential for broader applications in healthcare, beyond just tumor dynamics prediction.

**Implications for Clinical Practice**: The results of this study have important implications for clinical practice, particularly in personalized medicine. The improved accuracy and generalization offered by hybrid models using autoencoder-extracted features suggest that these methods could enhance decision-making processes in the treatment of patients undergoing GKRS. For example, more accurate predictions of tumor progression could inform decisions on whether to pursue aggressive treatments or focus on palliative care, thus optimizing patient outcomes [54]. Moreover, the potential overfitting observed in models like Extra Trees highlights the need for careful model selection and validation in clinical settings. Overfitting can lead to overly optimistic performance metrics that do not generalize well to new patients, which could have serious consequences in a medical context. Therefore, it is essential to prioritize models that balance high performance with the ability to generalize effectively [55].

**Dataset Biases and Limitations**: In analyzing the results, it is important to consider potential biases and limitations related to the dataset. Key factors include:

*Dataset Size*: A smaller dataset may not fully represent the variability of the broader population, potentially limiting the generalizability of the findings. Statistical power may be compromised, which could affect the reliability of the conclusions drawn.
*Dataset Composition*: The composition of the dataset can introduce biases. If the dataset is not representative of the population being studied (e.g., due to demographic imbalances or selection biases), the results may not accurately reflect the true characteristics or behaviors of the broader group.
*Sampling Methods*: The methods used to collect and sample data may also impact the results. Any inherent biases in the sampling process can affect the overall validity of the analysis.
*Data Quality*: Variations in data quality, such as inconsistencies, missing values, or errors, can influence the outcomes and interpretations of the study.

By acknowledging these limitations, we can better understand the scope of the results and consider areas for further research or data collection to address these issues.

**Limitations and Future Directions**: One limitation of this study is the risk of overfitting, particularly in models such as Extra Trees, which showed perfect classification in some scenarios. This suggests that while such models may perform exceptionally well on training data, they may not generalize as effectively to unseen data. Future research should explore additional regularization techniques or alternative model architectures that mitigate this risk [56]. Additionally, while autoencoders improve model performance, they can introduce complexity and reduce interpretability. This is a critical consideration in clinical settings, where model transparency is crucial for adoption by healthcare professionals [57].

**Clinical Implications of Improved Accuracy in Predicting Tumor Dynamics** Improving the accuracy of predicting tumor dynamics has significant clinical implications. Enhanced predictive models can lead to:

*Personalized Treatment Plans***:** More accurate predictions enable tailored treatment strategies based on individual tumor behavior and growth patterns. This personalization can optimize therapeutic outcomes and minimize unnecessary treatments.
*Early Intervention***:** By predicting tumor progression more reliably, clinicians can identify high-risk patients earlier, allowing for timely interventions and potentially improving overall survival rates.
*Optimized Resource Allocation***:** Accurate predictions help in better planning and resource management, reducing the costs associated with ineffective treatments and hospital stays.
*Enhanced Patient Counseling***:** Reliable predictions provide patients with clearer expectations about their prognosis, helping them make informed decisions about their treatment and care.

**Exploring Additional Variables for Enhanced Predictive Models** To further enhance predictive models, incorporating additional variables could be beneficial:

*Genetic Markers*: Integrating genetic markers into predictive models can offer deeper insights into tumor behavior and patient-specific risk factors. Genetic information may reveal predispositions to aggressive tumor types or responses to specific therapies.
*Molecular and Omics Data*: Including molecular data (e.g., proteomics, metabolomics) can improve model accuracy by capturing the complex biological interactions within tumors.
*Patient-Reported Outcomes*: Factors such as symptom profiles and quality of life indicators can provide valuable context for predicting tumor dynamics and patient responses.
*Imaging Data*: Advanced imaging techniques, such as radiomics, could offer additional information about tumor characteristics and changes over time.

By exploring and integrating these variables, predictive models can become more robust and applicable to a wider range of clinical scenarios, ultimately leading to improved patient outcomes and more effective treatment strategies.

Finally, this study focused on the prediction of tumor dynamics post-GKRS, a specific clinical application. Future research should investigate whether the benefits of these hybrid ML models extend to other medical domains. Expanding the scope of application could validate the broader utility of these techniques in various aspects of healthcare, potentially leading to more widespread adoption in clinical practice [58,59,60,61,62,63].

## 4. Conclusions

The integration of deep learning autoencoders with traditional ML models offers a promising approach to improving the accuracy and reliability of clinical predictions, particularly in complex scenarios such as predicting tumor dynamics following GKRS. This study contributes to the growing body of evidence supporting hybrid ML techniques in clinical data analysis and highlights the importance of further exploration to enhance patient outcomes across various medical fields.

## Figures and Tables

**Table 1:** Patient demographics for brain metastases from various primary cancers.

Category	Characteristics	Value
(S)	Age (yr)	
	Median (range)	64(39–85)
(S)	Sex	
	Male # (%)	45(58.44%)
	Female # (%)	32(41.56%)
	C1yr—control over one year (cm^3^)	17 *
	Median (range)	0.9(0–30)
	Patience with extra cranial MTS	6 *
		54
(T)	Receiving pre-treatment, systemic treatment	68
	Deceased before 1 year	1 *
		25
(C)	KPS score	9 *
	100	26 (33.77%)
	90	8 (10.39%)
	80	22 (28.57%)
	70	12 (15.58%)
	The number of lesions	
	Median(range)	2 (1–30)
	1–3	52
	4–6	12
	7–10	8
	>10	5
(R)	Beam on time on V1 (min/cm^3^)	
	Median(range)	0.82 (0.47–2.33)
(R)	Beam on time on V2 (min/cm^3^)	1 *
	Median(range)	0.83 (0.60–3.00)
(R)	Beam on time on V3 (min/cm^3^)	2 *
	Median(range)	0.83 (0.46–4.00)
	Total tumor volume (# of patients with):	
	<5 cm^3^	34
	<=10 cm^3^	13
	>10 cm^3^	30
(R)	Tumor dynamics (# of patients with):	
	- Progression	6
	- Regression	71

S, socio-demographic; C, clinical; T, treatment; R, radiosurgery; * number of missing data; # = number of.

**Table 2:** Top lazy predict models with default parameters.

Model	Accuracy	Balanced Accuracy	ROC AUC	F1 Score	Time Taken
SVC	1	1	1	1	0.02
ExtraTreesClassifier	1	1	1	1	0.15
KNeighborsClassifier	0.97	0.97	0.97	0.97	0.04
LogisticRegression	0.94	0.95	0.95	0.94	0.03
XGBClassifier	0.94	0.95	0.95	0.94	0.05
RandomForestClassifier	0.92	0.92	0.92	0.92	0.19

**Table 3:** Search space for tuning hyperparameter values.

Model	Parameters	Grid Search Space
SVC	C	0.1, 1, 10, 100
	gamma	scale, auto
	kernel	linear, rbf
KNeighborsClassifier	n_neighbors	3, 5, 7, 10
	weights	uniform
	*p*	1, 2
ExtraTreesClassifier	n_estimators	100, 200
	min_samples_split	2
	max_depth	None, 10
	min_samples_leaf	1
	max_features	sqrt
LogisticRegression (LR)	penalty	11, 12
	solver	liblinear
	C	0.001, 0.01, 0.1, 1, 10, 100
	max_iter	1000, 5000
XGBClassifier	n_estimators	100, 200, 300
	learning_rate	0.01, 0.05, 0.1
	max_depth	3, 5
	min_child_weight	1
	subsample	0.8
	colsample_bytree	0.8
	gamma	0
RandomForestClassifier	n_estimators	10, 50, 100, 200
	max_depth	None, 10, 20, 30
	min_samples_split	2, 5
	min_samples_leaf	1, 2

**Table 4:** Classification report for the six traditional ML models tested, with hyperparameter tuning.

Classification Report for Logistic Regression:				
	precision	recall	F1-score	Support
0	1	0.96	0.98	24
1	0.96	1	0.98	23
accuracy			0.98	47
macro avg	0.98	0.98	0.98	47
weighted avg	0.98	0.98	0.98	47
Classification Report for SVM:				
	precision	recall	F1-score	Support
0	0.96	0.92	0.94	24
1	0.92	0.96	0.94	23
accuracy			0.94	47
macro avg	0.94	0.94	0.94	47
weighted avg	0.94	0.94	0.94	47
Classification Report for KNN:				
	precision	recall	F1-score	Support
0	0.95	0.88	0.91	24
1	0.88	0.96	0.92	23
accuracy			0.91	47
macro avg	0.92	0.92	0.91	47
weighted avg	0.92	0.91	0.91	47
Classification Report for Extra Trees:				
	precision	recall	F1-score	Support
0	1	1	1	24
1	1	1	1	23
accuracy			1	47
macro avg	1	1	1	47
weighted avg	1	1	1	47
Classification Report for Random Forest:				
	precision	recall	F1-score	Support
0	1	0.92	0.96	24
1	0.92	1	0.96	23
accuracy			0.96	47
macro avg	0.96	0.96	0.96	47
weighted avg	0.96	0.96	0.96	47
Classification Report for XGBoost:				
	precision	recall	F1-score	Support
0	1	0.96	0.98	24
1	0.96	1	0.98	23
accuracy			0.98	47
macro avg	0.98	0.98	0.98	47
weighted avg	0.98	0.98	0.98	47

**Table 5:** Accuracy and AUC for the six traditional ML models tested, with hyperparameter tuning.

Model	Accuracy	AUC
Logistic Regression	0.979	0.98
SVM	0.936	0.94
KNN	0.915	0.92
Extra Trees	0.979	0.98
Random Forest	1.000	1.00
XGBoost	0.979	0.98

**Table 6:** Classification report for the six models tested using the Encoder from the AE without compression and with compression (the results in parenthesis) in the bottleneck layer.

Classification Report for Logistic Regression:				
	precision	recall	F1-score	Support
0	1 (1)	0.88 (0.83)	0.93 (0.91)	24
1	0.88 (0.85)	1 (1)	0.94 (0.92)	23
accuracy			0.94 (0.91)	47
macro avg	0.94 (0.93)	0.94 (0.92)	0.94 (0.91)	47
weighted avg	0.94 (0.93)	0.94 (0.91)	0.94 (0.91)	47
Classification Report for SVM:				
	precision	recall	F1-score	Support
0	0.95 (1)	0.75 (0.92)	0.84 (0.96)	24
1	0.79 (0.92)	0.96 (1)	0.86 (0.96)	23
accuracy			0.85 (0.96)	47
macro avg	0.87 (0.96)	0.85 (0.96)	0.85 (0.96)	47
weighted avg	0.87 (0.96)	0.85 (0.96)	0.85 (0.96)	47
Classification Report for KNN:				
	precision	recall	F1-score	Support
0	0.95 (1)	0.79 (0.83)	0.86 (0.91)	24
1	0.81 (0.85)	0.96 (1)	0.88 (0.92)	23
accuracy			0.87 (0.91)	47
macro avg	0.88 (0.93)	0.87 (0.92)	0.87 (0.91)	47
weighted avg	0.88 (0.93)	0.87 (0.91)	0.87 (0.91)	47
Classification Report for Extra Trees:				
	precision	recall	F1-score	Support
0	0.96 (0.96)	0.96 (1)	0.96 (0.98)	24
1	0.96 (1)	0.96 (0.96)	0.96 (0.98)	23
accuracy			0.96 (0.98)	47
macro avg	0.96 (0.98)	0.96 (0.98)	0.96 (0.98)	47
weighted avg	0.96 (0.98)	0.96 (0.98)	0.96 (0.98)	47
Classification Report for Random Forest:				
	precision	recall	F1-score	Support
0	0.96 (1)	0.92 (0.96)	0.94 (0.98)	24
1	0.92 (0.96)	0.96 (1)	0.94 (0.98)	23
accuracy			0.94 (0.98)	47
macro avg	0.94 (0.98)	0.94 (0.98)	0.94 (0.98)	47
weighted avg	0.94 (0.98)	0.94 (0.98)	0.94 (0.98)	47
Classification Report for XGBoost:				
	precision	recall	F1-score	Support
0	0.96 (0.96)	0.92 (0.92)	0.94 (0.94)	24
1	0.92 (0.92)	0.96 (0.96)	0.94 (0.94)	23
accuracy			0.94 (0.94)	47
macro avg	0.94 (0.94)	0.94 (0.94)	0.94 (0.94)	47
weighted avg	0.94 (0.94)	0.94 (0.94)	0.94 (0.94)	47

## Data Availability

The raw data supporting the conclusions of this article will be made available by the authors on request.

## References

[1] Lopez A.D., Mathers C.D., Ezzati M., Jamison D.T., Murray C.J. (2006). Global and regional burden of disease and risk factors, 2001: Systematic analysis of population health data. Lancet.

[2] Lamba N., Wen P.Y., Aizer A.A. (2021). Epidemiology of brain metastases and leptomeningeal disease. Neuro-Oncol..

[3] Soffietti R., Rudā R., Mutani R. (2002). Management of brain metastases. J. Neurol..

[4] Gavrilovic I.T., Posner J.B. (2005). Brain metastases: Epidemiology and pathophysiology. J. Neurooncol..

[5] Posner J.B., Chernik N.L. (1978). Intracranial metastases from systemic cancer. Adv. Neurol..

[6] Schouten J.P., McElgunn C.J., Waaijer R., Zwijnenburg D., Diepvens F., Pals G. (2002). Relative quantification of 40 nucleic acid sequences by multiplex ligation-dependent probe amplification. Nucleic Acids Res..

[7] Sacks P., Rahman M. (2020). Epidemiology of Brain Metastases. Neurosurg. Clin. N. Am..

[8] Noh T., Walbert T., Schiff D., van den Bent M.J. (2018). Chapter 6—Brain metastasis: Clinical manifestations, symptom management, and palliative care. Handbook of Clinical Neurology.

[9] Dasgupta A., Maitre M., Pungavkar S., Gupta T. (2022). Magnetic Resonance Imaging in the Contemporary Management of Medulloblastoma: Current and Emerging Applications. Methods Mol. Biol..

[10] Suh J.H., Kotecha R., Chao S.T., Ahluwalia M.S., Sahgal A., Chang E.L. (2020). Current approaches to the management of brain metastases. Nat. Rev. Clin. Oncol..

[11] Pérez-Larraya J.G., Hildebrand J., Biller J., Ferro J.M. (2014). Chapter 77—Brain metastases††This chapter is dedicated to the memory of Professor Jerzy Hildebrand. Handbook of Clinical Neurology.

[12] Gerosa M., Nicolato A., Foroni R., Zanotti B., Tomazzoli L., Miscusi M., Alessandrini F., Bricolo A. (2002). Gamma knife radiosurgery for brain metastases: A primary therapeutic option. J. Neurosurg..

[13] Hirway S.U., Weinberg S.H. (2022). A review of computational modeling, machine learning and image analysis in cancer metastasis dynamics. Comput. Syst. Oncol..

[14] Uzunova H., Schultz S., Handels H., Ehrhardt J. (2019). Unsupervised pathology detection in medical images using conditional variational autoencoders. Int. J. Comput. Assist. Radiol. Surg..

[15] Segato A., Marzullo A., Calimeri F., De Momi E. (2020). Artificial Intelligence for Brain Diseases: A Systematic Review. APL Bioeng..

[16] Basu K., Sinha R., Ong A., Basu T. (2020). Artificial Intelligence: How Is It Changing Medical Sciences and Its Future?. Indian J. Dermatol..

[17] Senders J.T., Staples P.C., Karhade A.V., Zaki M.M., Gormley W.B., Broekman M.L.D., Smith T.R., Arnaout O. (2018). Machine Learning and Neurosurgical Outcome Prediction: A Systematic Review. World Neurosurg..

[18] Yang Y.J., Bang C.S. (2019). Application of Artificial Intelligence in Gastroenterology. World J. Gastroenterol..

[19] Panesar S.S., D’Souza R.N., Yeh F.-C., Fernandez-Miranda J.C. (2019). Machine Learning Versus Logistic Regression Methods for 2-Year Mortality Prognostication in a Small, Heterogeneous Glioma Database. World Neurosurg. X.

[20] Marcus J.L., Sewell W.C., Balzer L.B., Krakower D.S. (2020). Artificial Intelligence and Machine Learning for HIV Prevention: Emerging Approaches to Ending the Epidemic. Curr. HIV/AIDS Rep..

[21] Vega-Huerta H., Villanueva-Alarcón R., Mauricio D., Moreno J.G., Vilca H.D.C., Rodriguez D., Rodriguez C. (2022). Convolutional Neural Networks on Assembling Classification Models to Detect Melanoma Skin Cancer. Int. J. Online Biomed. Eng. IJOE.

[22] Uspenskaya-Cadoz O., Alamuri C., Wang L., Yang M., Khinda S., Nigmatullina Y., Cao T., Kayal N., O’Keefe M., Rubel C. (2019). Machine Learning Algorithm Helps Identify Non-Diagnosed Prodromal Alzheimer’s Disease Patients in the General Population. J. Prev. Alzheimers Dis..

[23] Ghafouri-Fard S., Taheri M., Omrani M.D., Daaee A., Mohammad-Rahimi H. (2020). Application of Artificial Neural Network for Prediction of Risk of Multiple Sclerosis Based on Single Nucleotide Polymorphism Genotypes. J. Mol. Neurosci. MN.

[24] McNemar Q. (1947). Note on the sampling error of the difference between correlated proportions or percentages. Psychometrika.

[25] Bonferroni C.E. (1936). Teoria statistica delle classi e calcolo delle probabilità. Pubbl. R Ist. Super. Di Sci. Econ. Commer. Di Firenze.

[26] Benjamini Y., Hochberg Y. (1995). Controlling the false discovery rate: A practical and powerful approach to multiple testing. J. R. Stat. Soc. Ser. B.

[27] LeCun Y. (1987). ModÈlesconnexionistes de L’apprentissage. Ph.D. Thesis.

[28] Bourlard H., Kamp Y. (1988). Auto-association by multilayer perceptrons and singular value decomposition. Biol. Cybern..

[29] Kramer M.A. (1991). Nonlinear principal component analysis using autoassociative neural networks. AIChE J..

[30] Hinton G.E., Zemel R.S. (1994). Autoencoders, minimum description length, and helmholtz free energy. Advances in Neural Information Processing Systems 6 (NIPS 1993).

[31] Courville A. (2012). Unsupervised feature learning and deep learning: A review and new perspectives. arXiv.

[32] https://en.wikipedia.org/wiki/Autoencoder.

[33] Brenner D.J. (2008). the linear-quadratic model is an appropriate methodology for determining isoeffective doses at large doses per fraction. Semin. Radiat. Oncol..

[34] Fowler J.F. (1989). The linear-quadratic formula and progress in fractionated radiotherapy. Br. J. Radiol..

[35] Higuchi Y., Serizawa T., Nagano O., Matsuda S., Ono J., Sato M., Iwadate Y., Saeki N. (2009). Three-staged stereotactic radiotherapy without whole brain irradiation for large metastatic brain tumors. Int. J. Radiat. Oncol. Biol. Phys..

[36] Rodríguez P., Bautista M.A., Gonzàlez J., Escalera S. (2018). Beyond one-hot encoding: Lower dimensional target embedding. Image Vis. Comput..

[37] Ahsan M.M., Mahmud M.A.P., Saha P.K., Gupta K.D., Siddique Z. (2021). Effect of Data Scaling Methods on Machine Learning Algorithms and Model Performance. Technologies.

[38] Rao A., Monteiro J.M., Mourao-Miranda J., Alzheimer’s Disease Initiative (2017). Predictive Modelling Using Neuroimaging Data in the Presence of Confounds. NeuroImage.

[39] Cramer H. (1946). Mathematical Methods of Statistics.

[40] Mukaka M. (2012). A Guide to Appropriate Use of Correlation Coefficient in Medical Research. Malawi Med. J. J. Med. Assoc. Malawi.

[41] Zychlinski S. Dython: A Set of Data Tools in Python. http://shakedzy.xyz/dython.

[42] Chawla N.V., Bowyer K.W., Hall L.O., Kegelmeyer W.P. (2002). SMOTE: Synthetic Minority Over-Sampling Technique. J. Artif. Intell. Res..

[43] Zhu T., Lin Y., Liu Y. (2017). Synthetic Minority Oversampling Technique for Multiclass Imbalance Problems. Pattern Recognit..

[44] Raschka S., Patterson J., Nolet C. (2020). machine learning in python: Main developments and technology trends in data science, machine learning, and artificial intelligence. Information.

[45] Bzdok D., Altman N., Krzywinski M. (2018). Statistics versus Machine Learning. Nat. Methods.

[46] Zhang Y., Yang Y. (2015). Cross-Validation for Selecting a Model Selection Procedure. J. Econom..

[47] Franklin J. (2005). The Elements of Statistical Learning: Data Mining, Inference and Prediction. Math. Intell..

[48] Hybrid Learning Systems: Integrating Traditional Machine Learning with Deep learning Techniques. https://www.researchgate.net/publication/380366289_Hybrid_Learning_Systems_Integrating_Traditional_Machine_Learning_with_Deep_learning_Techniques.

[49] Baldi P. Autoencoders, unsupervised learning, and deep architectures. Proceedings of the ICML Workshop on Unsupervised and Transfer Learning.

[50] Litjens G., Kooi T., Bejnordi B.E., Setio AA A., Ciompi F., Ghafoorian M., van der Laak J.A.W.M., van Ginneken B., Sánchez C.I. (2017). A survey on deep learning in medical image analysis. Med. Image Anal..

[51] Esteva A., Robicquet A., Ramsundar B., Kuleshov V., DePristo M., Chou K., Cui C., Corrado G., Thrun S. (2019). A guide to deep learning in healthcare. Nat. Med..

[52] Chen T., Guestrin C. XGBoost: A scalable tree boosting system. Proceedings of the 22nd ACM SIGKDD International Conference on Knowledge Discovery and Data Mining.

[53] Lundberg S.M., Nair B., Vavilala M.S., Horibe M., Eisses M.J., Adams T., Liston D.E., Low D.K.-W., Newman S.-F., Kim J. (2018). Explainable machine learning predictions for the prevention of hypoxaemia during surgery. Nat. Biomed. Eng..

[54] Sahiner B., Pezeshk A., Hadjiiski L.M., Wang X., Drukker K., Cha K.H., Summers R.M., Giger M.L. (2019). Deep learning in medical imaging and radiation therapy. Med. Phys..

[55] Sanchez-Martinez S., Camara O., Piella G., Maja C., Gonzales-Ballester M.Á., Miron M., Alfredo V., Gómez E., Fraser A.G., Bijnens B. (2022). Machine Learning for Clinical Decision-Making: Challenges and Opportunities in Cardiovascular Imaging. Front. Cardiovasc. Med..

[56] Dietterich T.G. (2000). Ensemble Methods in Machine Learning. Multiple Classifier Systems.

[57] Rudin C. (2019). Stop explaining black box machine learning models for high-stakes decisions and use interpretable models instead. Nat. Mach. Intell..

[58] Rajkomar A., Dean J., Kohane I. (2019). Machine learning in medicine. N. Engl. J. Med..

[59] Yousefi O., Azami P., Sabahi M., Dabecco R., Adada B., Borghei-Razavi H. (2022). Management of Optic Pathway Glioma: A Systematic Review and Meta-Analysis. Cancers.

[60] Kazempour A., Balogh P. (2024). Margination behavior of a circulating cell in a tortuous microvessel. Phys. Fluids.

[61] Fadavi N., Fadavi N. (2024). Early Recognition of Parkinson’s Disease through Acoustic Analysis and Machine Learning. arXiv.

[62] Ashrafi N., Liu Y., Xu X., Wang Y., Zhao Z., Pishgar M. (2024). Deep learning model utilization for mortality prediction in mechanically ventilated ICU patients. Inform. Med. Unlocked.

[63] Ashrafi N., Abdollahi A., Pishgar M. (2024). Enhanced Prediction of Ventilator-Associated Pneumonia in Patients with Traumatic Brain Injury Using Advanced Machine Learning Techniques. arXiv.

